# Direct and indirect effects of the COVID-19 pandemic on mortality: an individual-level population-scale analysis using linked electronic health records, Wales, United Kingdom, 2016 to 2022

**DOI:** 10.2807/1560-7917.ES.2024.29.50.2400085

**Published:** 2024-12-12

**Authors:** Rhiannon K Owen, James D van Oppen, Jane Lyons, Ashley Akbari, Gareth Davies, Fatemeh Torabi, Keith R Abrams, Ronan A Lyons

**Affiliations:** 1 Population Data Science, Swansea University Medical School, Swansea University, Swansea, United Kingdom; 2 Centre for Urgent and Emergency Care Research (CURE), University of Sheffield, Sheffield, United Kingdom; 3 Department of Statistics & Warwick Medical School (WMS), University of Warwick, Coventry, United Kingdom

**Keywords:** COVID-19, Epidemiology, Healthcare Disparities, Public health, Socioeconomic Factors

## Abstract

**Background:**

The COVID-19 pandemic resulted in increased mortality directly and indirectly associated with COVID-19.

**Aim:**

To assess the impact of the COVID-19 pandemic on all-cause and disease-specific mortality and explore potential health inequalities associated with area-level deprivation in Wales.

**Methods:**

Two population-based cohort studies were derived from multi-sourced, linked demographic, administrative and electronic health record data from 2016 to 2019 (n = 3,113,319) and 2020 to 2022 (n = 3,571,471). Data were analysed using generalised linear models adjusting for age, sex, area-level deprivation and time at risk.

**Results:**

COVID-19 deaths peaked in January 2021 (54.9/100,000 person-months, 95% confidence interval (CI): 52.4–57.5). The pandemic indirectly affected deaths, with higher than expected maximum relative mortality rates (RR) related to cancer (RR: 1.24, 95% CI: 1.13–1.36), infectious diseases (excluding respiratory infections) (RR: 2.09, 95% CI: 1.27– 3.43), circulatory system (RR: 1.41, 95% CI: 1.28–1.56), trauma (RR: 2.04, 95% CI: 1.57– 2.65), digestive system (RR: 1.54, 95% CI: 1.25–1.91), nervous system (RR: 1.63; 95% CI: 1.34–2.00) and mental and behavioural disorders (RR: 1.85, 95% CI: 1.58–2.16). Mortality associated with respiratory diseases (unrelated to COVID-19) were lower than expected (minimum RR: 0.52, 95% CI: 0.45–0.60). All-cause mortality was lower in least deprived communities compared with most deprived (RR: 0.61, 95% CI: 0.60–0.62), and the magnitude of this effect increased during the pandemic.

**Conclusions:**

All-cause and disease-specific mortality directly and indirectly associated with COVID-19 increased during the COVID-19 pandemic. Socioeconomic disparities were exacerbated during this time.

**Key public health message**

**What did you want to address in this study and why?**

The COVID-19 pandemic resulted in increased all-cause mortality. There is a paucity of evidence exploring the impact of socioeconomic status on the direct and indirect effects of the pandemic. We aimed to assess excess all-cause and disease-specific mortality arising from the pandemic and identify potential health inequalities related to area-level deprivation in Wales.

**What have we learnt from this study?**

Using data from 2016 to 2022, we found that during the pandemic, excess deaths were observed from all-causes including COVID-19, infectious diseases, cancers, circulatory diseases, diseases of digestive and nervous systems, mental disorders and trauma in Wales. Respiratory deaths (excluding COVID-19) were lower than expected. Socioeconomic disparities were exacerbated during the pandemic.

**What are the implications of your findings for public health?**

The findings of this study could be used to inform further research to understand the aetiology of continued excess deaths and health inequalities in Wales, with a view to inform healthcare policy and implement preventative measures.

## Introduction

The COVID-19 pandemic was declared a public health emergency of international concern on 30 January 2020 [1] and lasted until 5 May 2023 [2]. During this time, nearly 7 million COVID-19 related deaths were reported to the World Health Organization (WHO) [3], with the true death toll estimated to be in excess of 18 million people [4]. The COVID-19 pandemic placed unprecedented pressure on healthcare systems globally [5], leading to reduced healthcare utilisation [6,7] as a result of reduced healthcare services and delivery [7,8] and poorer health outcomes [7]. Following the pandemic, countries across the world have reported ongoing excess deaths from all causes [9]. A substantial proportion of these deaths are likely to be a result of indirect consequences of the pandemic including delayed diagnoses and/or delayed treatment of conditions [10]. These consequential deaths are referred to as indirect effects.

Studies [9,11] have evaluated excess deaths by comparing the number of all-cause or disease-specific deaths over a given time period to the expected number of deaths based on predictions from historical trends. The majority of studies have evaluated excess deaths using aggregated data at a population level. This approach can be prone to aggregation bias when exploring associations with individual-level characteristics such as age, sex, ethnicity and socioeconomic factors [12]. The impact of racial and ethnic disparities on the indirect effects of COVID-19 has been widely explored in the literature [13,14] and demonstrates an excess mortality among racial/ethnic minority groups. However, while it has been suggested that the indirect effects of the COVID-19 pandemic may also have disproportionately impacted those from lower socioeconomic backgrounds, there is a paucity of supporting evidence [7].

In this study, we used linked electronic health records to quantify the direct and indirect effects of the COVID-19 pandemic on all-cause and disease-specific mortality, accounting for competing causes of death at the individual level for the population of Wales. We further investigated the impact of potential health inequalities related to socioeconomic status on all-cause and disease-specific mortality.

## Methods

### Participants and setting

Two population-level cohorts (C16 and C20) were created using multi-sourced administrative, demographic and healthcare data held within the Secure Anonymised Information Linkage (SAIL) Databank (www.saildatabank.com) [15]. The C16 and C20 cohorts contain linked data at the individual participant level using anonymised linkage fields. Full information regarding the curation of the C16 and C20 cohorts and associated methodology have been reported elsewhere [16]. The C16 cohort provides comparative data on population mortality rates and can be used to predict (i.e. calculate expected) mortality for comparison with observed mortality in the C20 cohort.

The C16 cohort included all residents in Wales on 1 January 2016 with follow-up until death, break in Welsh residency, or 31 December 2019. The C16 cohort did not capture individuals migrating to, or born in, Wales between 2016 to 2019, and therefore for analyses involving trends of mortality rates, the population was assumed to remain constant from 1 January 2016 to 31 December 2019.

The C20 cohort included all individuals resident in Wales from 1 January 2020 with follow-up until death, break in Welsh residency, or cohort end (31 December 2022). The C20 cohort was updated to include individuals migrating to, or born in, Wales after 1 January 2020.

### Data sources

Demographic data were obtained from the Welsh Demographic Service Dataset (WDSD), which holds administrative information for the population of Wales known to the National Health Service (NHS). Mortality data were obtained from the Annual District Death Extract (ADDE) from the Office for National Statistics (ONS), which holds information regarding the dates and causes of death for all Welsh residents (including those who died outside of Wales). For the C20 cohort, two COVID-19 data sources were added and updated daily to support rapid analysis: the COVID-19 Consolidated Death Data Source (CDDS) created by Digital Health and Care Wales (DHCW) - formerly known as the NHS Wales Informatics Service (NWIS), and the Annual District Death Daily (ADDD) dataset from the ONS. Demographic and mortality data were linked to residential data to identify Lower-layer Super Output Area (LSOA) version 2011. These were used to assign area-level deprivation from 1 (most deprived) to 5 (least deprived) using the Welsh Index of Multiple Deprivation version 2019 (WIMD) [17].

### Outcome measures

The primary outcome measure was all-cause mortality. Secondary outcomes were disease-specific mortality grouped by International Classification of Disease tenth revision (ICD-10) chapter version 2016 [18]. The ICD-10 codes for each chapter are provided in the Table. Deaths from COVID-19 were identified using ICD-10 codes U071, U072, B972, U049, Z038 and Z115. Mortality was primarily identified using the underlying cause of death recorded in ADDE from the ONS, and supplemented by CDDS, WDSD and ADDD in that order.

**Table:** Cohort characteristics, Wales, 2016–2022 (n = 6,684,790)

Characteristics	C16 cohort (2016-2019)		C20 cohort (2020–2022)	
	n	%	n	%
Total	3,113,319	NA	3,571,471	NA
All-cause deaths	119,201	3.8	109,735	3.1
Mortality rate per 100,000 person years	957.2	NA	1,024.2	NA
Age (median, IQR)	42	22–60	40	22–60
Missing	0	0.0	0	0.0
Sex				
Female	1,560,206	50.1	1,787,198	50.0
Male	1,553,113	49.9	1,784,273	50.0
Missing	0	0.0	0	0.0
WIMD				
1 – Most deprived	631,648	20.3	679,701	19.0
2	619,494	19.9	657,708	18.4
3	625,620	20.1	667,833	18.7
4	616,888	19.8	649,634	18.2
5 – Least deprived	619,669	19.9	654,193	18.4
Missing	0	0.0	262,402	7.3
ICD-10 chapter (ICD-10 codes)^a^				
1: Infectious and parasitic diseases (A00–B99)	1,226	1.0	1,039	1.0
2: Neoplasms (C00-D48)	33,837	28.4	27,565	25.1
3: Blood and blood forming organs (D50-D89)	237	0.2	192	0.2
4: Endocrine, nutritional, and metabolic (E00-E90)	1,824	1.5	1,812	1.7
5: Mental and behavioural disorders (F00-F99)	9,423	7.9	8,275	7.5
6: Nervous system (G00-G99)	5,186	4.4	6,060	5.5
9: Circulatory system (I00-I99)	30,445	25.5	25,789 (	23.5
10: Respiratory system (J00-J99)	18,369	15.4	12,353	11.3
11: Digestive system (K00-K93)	6,094	5.1	5,530	5.0
12: Skin and subcutaneous tissue (L00-L99)	482	0.4	395	0.4
13: Musculoskeletal system (M00-M99)	795	0.7	667	0.6
14: Genitourinary system (N00-N99)	2,254	1.9	1,823	1.7
17: Congenital malformations and chromosomal abnormalities (Q00-Q99)	249	0.2	233	0.2
18: Abnormal clinical and laboratory findings (R00-R99)	2,356	2.0	2,749	2.5
20: External causes of morbidity and mortality (V01-Y98)	4,391	3.7	4,089	3.7
22: Codes for special purposes (U00-U99)	88	0.1	9,399	8.6
Other	15	0.0	18	0.0
Missing	1,930	1.6	1,747	1.6

ICD-10: International Classification of Disease tenth revision [ref]; IQR: interquartile range; NA: not applicable; WIMD: Welsh Index of Multiple Deprivation version 2019.

^a^ Actuarial percentage calculated as the percentage of those who died.

### Statistical analysis

Trends in all-cause and disease-specific mortality were expressed as the mortality rate per 100,000 person-months together with their corresponding 95% confidence intervals (CIs). Predicted mortality rates were calculated to compare observed vs expected mortality. Negative binomial regression models, using an offset for population at risk, were fitted to monthly aggregated data in the C16 cohort accounting for trends using epidemiological month and seasonality using Fourier terms across years, and used to predict expected mortality in the C20 cohort. Outputs were visualised by plotting the observed vs expected mortality counts each month, highlighting where the observed counts are above (red) or below (green) the 95% prediction intervals obtained from the regression model. Prediction intervals were calculated using the upper and lower bounds of the CI in order to obtain quantiles of the negative binomial distribution, the minimum and maximum of which provided the lower and upper limits of the prediction interval [19]. Relative mortality rates (RR) were calculated as the ratio of observed and expected mortality rates with corresponding 95% CIs.

Zero-inflated Poisson regression models adjusted for age (centred at 50 years), sex (male/female), and WIMD were fitted at the individual participant level to assess the association between cohort and all-cause mortality, using an offset for time at risk [20]. Zero-inflated models were used to account for the high proportion of individuals alive in the cohort. Area-level deprivation was explored adding a main effect for WIMD and an interaction term for cohort and WIMD. Generalised linear models were assessed for overdispersion and model fit. Results were reported as relative risks and corresponding 95% CIs.

Multinomial regression models adjusted for age (centred at 50 years), sex (male/female) and area-level deprivation using WIMD were fitted at the individual participant level to assess the association between cohort and disease-specific mortality, including an offset for time at risk [20]. This approach accounts for death due to other reasons as a potentially competing risk. Cause-specific deaths of interest were neoplasms, endocrine, nutritional and metabolic diseases, mental and behavioural disorders, diseases of the nervous system, diseases of the circulatory system, diseases of the respiratory system, diseases of the digestive system, external causes and special purposes. Other causes of death were used as the reference group for comparison. Results were reported as odds ratios (OR) and corresponding 95% CIs.

Sensitivity analyses were undertaken in individuals aged 18 years and over to account for potential differences in the populations at risk owing to the C16 cohort not capturing births in Wales between 1 January 2016 and 31 December 2019.

Complete case analyses were undertaken, given the small proportion of missing data for covariates. All statistical analyses were performed using R software version 4.1.3 [21].

## Results

At cohort start, there were 3,113,319 individuals in C16 and 3,571,471 individuals in C20. The Table shows demographic and other characteristics for the C16 and C20 cohorts. The C16 cohort had a mean follow-up of 12,441,500 person-years and C20 had a mean follow-up of 9,748,397 person-years. During follow-up (4 years for C16 and 3 years for C20), the proportion of all-cause mortality was 3.8% and 3.1%, equivalent to a mortality rate of 957.2 and 1,024.2 per 100,000 person-years, respectively.

The population of Wales remained relatively consistent between C16 and C20 with 50.1% female in C16 and 50.0% female in C20. The C20 cohort was slightly younger with a median age of 40 years (interquartile range (IQR): 22–60) compared with a median age of 42 years (IQR: 22–60) in C16. Deprivation remained unchanged with ca 20% of the population in the most and least deprived quintiles. However, there was an increased proportion of missing data for deprivation status owing to missing LSOA for individuals in the C20 cohort (7.3%) compared with the C16 cohort (0%). Overall, missing data was low in both C16 and C20 with an actuarial percentage of 1.6% of individuals with a missing cause of death in both cohorts. Of those who died in the C20 cohort, 8.6% of deaths were recorded with codes used for special purposes. The rate of all-cause and COVID-specific deaths from 2016 to 2022 are provided in Supplementary Figure S1. The rate of COVID-19 related deaths peaked in April 2020 (48.9/100,000 person-months, 95% CI: 46.5–51.3) and January 2021 (54.9/100,000 person-months, 95% CI: 52.4–57.5) before plateauing.

### Observed vs expected all-cause mortality

**Figure 1:**
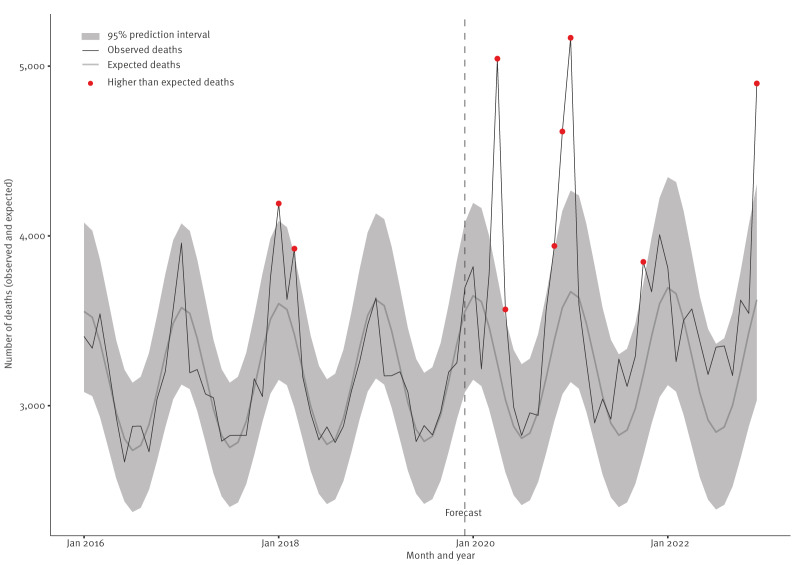
Observed versus expected all-cause mortality obtained from negative binomial regression models, Wales, 2016–2022 (n = 6,684,790)

Figure 1 illustrates the observed vs expected all-cause mortality over time. The highest observed vs expected relative mortality rate occurred in April 2020 where there appeared to be a 49% increased risk of death compared with that expected (RR: 1.49, 95% CI: 1.42–1.55) (Figure 1). Prior to the COVID-19 pandemic, there appeared to be a higher than expected mortality rate in January 2018, with 16% increased risk of death compared with that expected (RR: 1.16, 95% CI: 1.11–1.22).

Vertical dashed line indicates where the C16 cohort ends and the forecasted trend for the C20 cohort.

**Figure 2:**
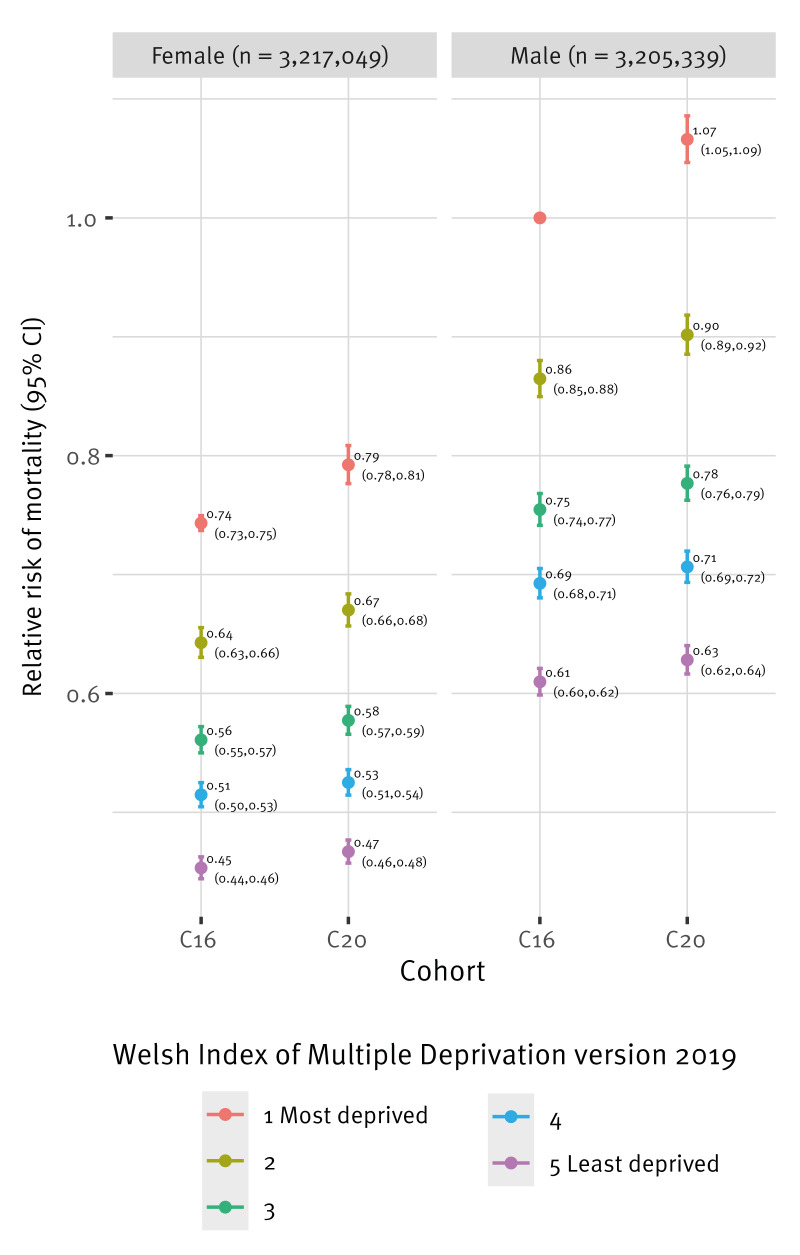
Relative risk of all-cause mortality by cohort, sex and Welsh Index of Multiple Deprivation obtained from zero-inflated Poisson regression models, Wales, 2016–2022 (n = 6,422,388)

Overall, the C20 cohort observed a 7% increased risk of all-cause mortality compared with the C16 cohort having adjusted for differences in age, sex and area-level deprivation (RR: 1.07, 95% CI: 1.05–1.09) (model coefficients are reported in Supplementary Table S1). Individuals in the least deprived communities had a 39% lower all-cause mortality compared with those in the most deprived communities (RR: 0.61, 95% CI: 0.60–0.62) (all-cause mortality rates by deprivation status is provided in Supplementary Figure S2). This socioeconomic disparity appeared to increase during the pandemic (Figure 2). For example, males in the most deprived groups had a significantly higher RR of death in C20 (RR: 1.07, 95% CI: 1.05–1.09) compared with males in the most deprived groups in C16. However, males in the least deprived group in C20 had a similar RR (RR: 0.63, 95% CI: 0.62–0.64), to males in the least deprived group in C16 (RR: 0.61, 95% CI: 0.60–­0.62), with overlapping 95% CIs suggesting no statistically significant difference. These findings were robust to sensitivity analyses including using adults aged 18 years and over only (model coefficients for adults only are provided in Supplementary Table S2).

CI: confidence interval.

C16 cohort: 2016–2019.

C20 cohort: 2020–2022.

### Disease-specific mortality

**Figure 3:**
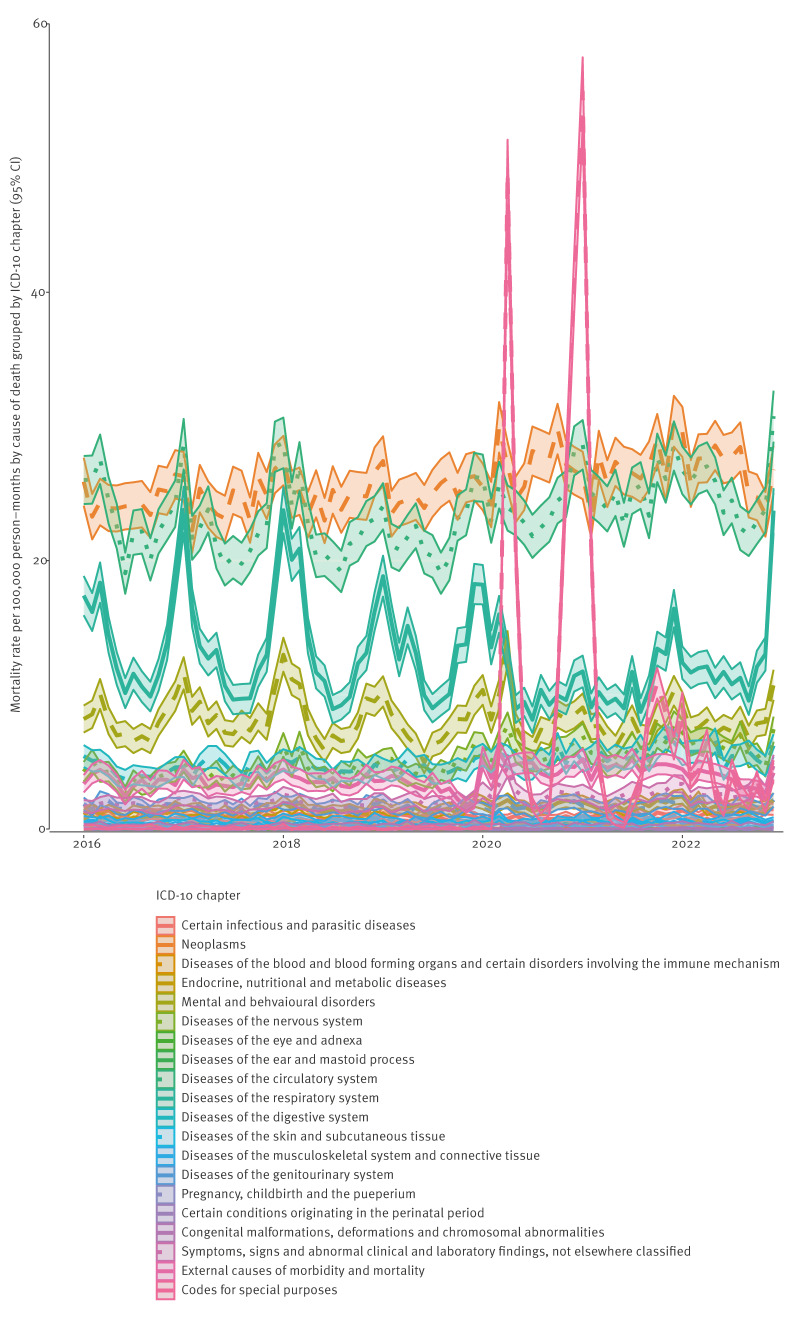
Trends and 95% confidence intervals in disease-specific mortality grouped by ICD-10 chapter, Wales, 2016–2022 (n = 6,684,790)

**Figure 4:**
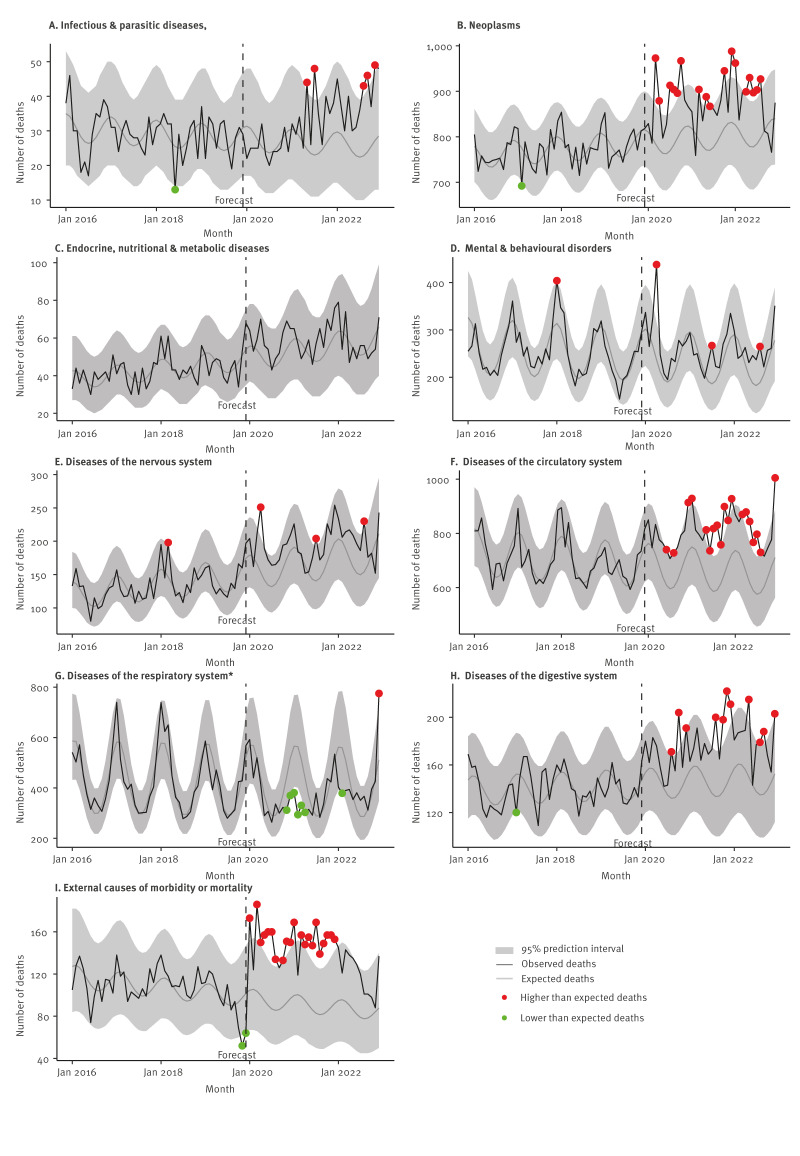
Observed versus expected disease-specific mortality by ICD-10 chapter from negative binomial regression models, Wales, 2016–2022 (n = 6,684,790)

Figure 3 illustrates trends from 2016 to 2022 in disease-specific mortality rates grouped by cause of death. The observed vs expected cause-specific mortality rates, adjusting for trend and seasonality, are presented in Figure 4. From 2020, deaths due to neoplasms (maximum RR in March 2020: 1.24, 95% CI: 1.13–1.36), circulatory disease (maximum RR in December 2022: 1.41, 95% CI: 1.28–1.56) and the digestive system (maximum RR in November 2021: 1.54, 95% CI: 1.25–1.91) were consistently higher than expected. Deaths due to respiratory diseases (excluding COVID-19) were mostly lower than expected (minimum RR in February 2021: 0.52, 95% CI: 0.45–0.60) until December 2022 (RR:1.52, 95% CI:1.36–1.69). Deaths due to endocrine, nutritional and metabolic diseases were within the expected range. Deaths due to mental and behavioural disorders (RR: 1.85, 95% CI: 1.58–2.16) and the nervous system (RR: 1.63, 95% CI: 1.34–2.00) were higher than expected and peaked during the first lockdown period in April 2020. Deaths due to infectious and parasitic diseases were higher than expected and peaked in July 2021 (RR: 2.09, 95% CI: 1.27–3.43). Deaths due to external causes were consistently higher than expected throughout 2021 and 2022 and also peaked in July 2021 (maximum RR in July 2021: 2.04, 95% CI: 1.57–2.65). The majority of deaths due to external causes during this period were as a result of falls (ICD-10 code: W00–W19), and intent to self-harm (ICD-10 code: X60–X84). Further investigation found that there was higher than expected mortality due to falls throughout 2021 and 2022 (observed versus expected number of deaths from falls are provided in Supplementary Figure S3). The highest peak was observed in December 2022 where individuals were four times as likely to die as result of a fall than expected (RR: 4.41, 95% CI: 2.68–7.29). The majority of secondary causes of deaths in individuals in C20 with falls as their primary cause of death were injuries to the head (ICD-10 code: S00–S09, 42%) and injuries to the hip and thigh (ICD-10 code: S70–S79, 25%). Deaths due to self-harm were as expected throughout 2020 to 2022 (observed versus expected number of deaths from self-harm are provided in Supplementary Figure S4).

Effect estimates obtained from multinomial models, accounting for competing causes of death, are provided in Supplementary Table S3. Compared with deaths from other causes, there appeared to be an 11% increased odds of death from neoplasms (OR: 1.11, 95% CI: 1.09–1.13), 31% increased odds of mortality from endocrine, nutritional and metabolic diseases (OR: 1.31, 95% CI: 1.23–1.39), 38% increased odds of mortality from diseases of the nervous system (OR: 1.38, 95% CI: 1.33– 1.43), 11% increased odds of mortality from diseases of the circulatory system (OR: 1.11, 95% CI: 1.09–1.13), 22% increased odds from diseases of the digestive system (OR: 1.22, 95% CI: 1.18–1.27) and 11% increased odds from external causes (OR: 1.11, 95% CI: 1.06–1.15) in the C20 cohort compared with C16 cohort. Compared with deaths from other causes, the C20 cohort had 12% decreased odds of mortality from diseases of the respiratory system excluding COVID-19 (OR: 0.88, 95% CI: 0.86–0.90) compared with C16, and 6% decreased odds of mortality due to mental and behavioural disorders (OR: 0.94, 95% CI: 0.92–0.97). There was 29% increased odds of survival compared with deaths from other causes in the C20 cohort compared with the C16 cohort (OR: 1.28, 95% CI: 1.28–1.30). The predicted probabilities of each cause of death by cohort, sex and age are provided in Supplementary Figure S5.

The odds of death from endocrine, nutritional and metabolic diseases, diseases of the respiratory system, diseases of the digestive system and deaths due to special codes decreased by 18% (OR: 0.82, 95% CI: 0.74–0.90), 24% (OR: 0.76, 95% CI: 0.73–0.79), 17% (OR: 0.83, 95% CI: 0.79–0.88) and 17% (OR: 0.83, 95% CI: 0.78–0.88), respectively, compared with deaths from other causes, for most deprived compared with least deprived communities (model coefficients are provided in Supplementary Table S3). However, the odds of death due to neoplasms, mental and behavioural disorders and diseases of the nervous system increased by 16% (OR: 1.16, 95% CI: 1.13–1.20), 15% (OR: 1.09, 95% CI: 1.09–1.20) and 65% (OR: 1.65, 95% CI: 1.56–1.75), respectively, compared with deaths from other causes, for most deprived compared with least deprived communities. Deaths due to diseases of the circulatory system and external causes did not appear to differ with deprivation. There was 89% increased odds of survival compared with deaths from other causes in individuals from least deprived compared with most deprived communities (OR: 1.89, 95% CI: 1.86–1.91). Results were robust to sensitivity analyses including adults only (model coefficients for adults only are provided Supplementary Table S4).

## Discussion

This study found that all-cause deaths were higher than expected during the COVID-19 pandemic in Wales. The rate of COVID-19 deaths peaked in April 2020 and January 2021. In addition to COVID-19, excess deaths were observed from infectious and parasitic diseases, cancers, circulatory disease, diseases of the digestive and nervous systems, mental and behavioural disorders and trauma. The majority of trauma-related deaths were as a result of falls and intent to self-harm.

Deaths due to respiratory diseases (excluding COVID-19) were consistently lower than expected until December 2022. This is consistent with previous analyses of morbidity in patients with chronic obstructive pulmonary disease (COPD) during lockdown in Scotland and Wales which showed a 48% reduction in primary care attendances and hospital admission. Suggested underlying causes were reduced transmission of respiratory infections overall, due to social distancing and reduced exposure to air pollution [22].

Increases in deaths due to cancers and digestive illnesses during the pandemic period may reflect their later identification due to personal or public messaging-inspired reluctance to attend healthcare settings, or possibly poorer diagnostic sensitivity due to increased healthcare delivery using remote consultation methods. Following diagnosis, delayed medical or surgical intervention due to hospital capacity and professional redeployments may also have contributed to higher mortality.

There appeared to be consistently higher (up to four times as many) fall-related deaths during the pandemic than expected. The majority of secondary causes of death in these individuals were recorded as injuries to the head and fractures to the hip and thigh. The higher fall-related deaths may be associated with frailty, based on older people with frailty having more frequent head injuries (due to unprotected falls) and poorer outcomes following fractures of the hip and thigh. The increase in fall-related deaths appeared to peak in December 2022. This suggests ongoing factors following the rapid decrease in COVID-19 related deaths. The increase in fall-related deaths during this time may be a result of delays in urgent care in Wales, including ambulance attendance and subsequent ramping, over-crowding in emergency departments [23] and delayed admissions [24]. Deaths due to femoral fractures may be attributable to delays in surgery and subsequent therapy, and/or change in incidence. Deaths directly due to head injuries and hip fractures may also suggest more proactive identification of frailty and delivery of palliative-focussed trauma care. Pandemic-associated loss of function and physiological resilience due to social isolation and disrupted management of chronic illnesses may have also caused increased susceptibility to falls. Similar increases in fall-related mortality during the pandemic have been reported in the United States and China [25,26].

It is likely that most of the excess deaths during the pandemic period were a result of several factors including changes in health-seeking behaviours during the pandemic [27], excess pressures placed on stretched health systems [28] and disrupted health service delivery [29]. Over the course of the pandemic period, several healthcare professions in Wales participated in industrial action which may have also adversely affected health service delivery and capacity. However, previous research has found that strike action does not result in excess mortality [30].

There appeared to be a disparity in all-cause mortality by area-level deprivation. Overall, individuals from most deprived communities had a higher risk of death compared with those from least deprived communities, and this disparity was exacerbated during the pandemic. However, there were higher odds of mortality from cancer, mental and behavioural disorders and diseases of the nervous system compared with deaths from other causes for least deprived individuals compared with most deprived. It is well-documented that these are considered more affluent diseases [31,32], and previous research has found that mortality rates for specific cancer types including breast [33], ovarian and prostate cancer [34] were higher in mid-least deprived areas of England and Wales compared with the most deprived areas. The models were adjusted to account for potential differences in age and sex across deprivation groups, so differences in mortality related to individuals in the least deprived communities potentially living longer and thus dying from diseases associated with ageing are unlikely to explain these findings. The discrepancy in cause of death between deprivation groups may be related to potential differences in access to care, education, lifestyle and/or living conditions.

Future work should aim to understand socioeconomic inequalities contributing to disparities in healthcare service and delivery needs. In particular, potential barriers to accessing and delivering emergency care services should be further explored to inform adequate provision. Further research is needed to understand potential differences in healthcare behaviours and access to healthcare services during the pandemic for individuals from different deprivation groups in Wales.

This study’s strength is its use of population-scale, individual-level, linked electronic health records to adjust for differences in age, sex, area-level deprivation and competing events of mortality at the individual-level, thereby reducing the risk of potential aggregation biases and providing unbiased estimates for cause of death. Further studies using population-scale linked electronic health records at the individual-level should be used in other countries to provide a basis for comparison to inform potential healthcare policy and decision-making at the system-level.

In this study, we were unable to capture individuals born in or migrating to Wales in the C16 cohort and thus we assumed that the C16 cohort remained constant. Sensitivity analyses were undertaken to explore the robustness of results to adults aged 18 years or older only. However, this analysis would not capture migration of adults to Wales. A large proportion of adults migrating to Wales are university students. It is unclear whether this would have an important impact on the analysis since during the COVID-19 pandemic many students remained at their family home and therefore may not have migrated into Wales in the C20 cohort. In the C20 cohort, 329,859 (10.2%) of the cohort were born in, or migrated to, Wales. However, the C20 cohort was younger (median 40 years) than C16 (median 42 years), and therefore may be healthier. This potential limitation is concerning since if this theory holds true, the study findings would underestimate the true indirect effects of the pandemic.

## Conclusion

The COVID-19 pandemic had a direct and indirect effect on mortality in Wales. Socioeconomic disparities were also exacerbated during this time. As communities and healthcare systems recover from COVID-19, it is essential to understand the aetiology of increased all-cause deaths and health inequalities.

## *Erratum

The title of Figure 4G was corrected on 13 December 2024.

## Supplementary Data

342000 Supplementary Material
