# Erratum for Euro Surveill. 2024;29(50)

**DOI:** 10.2807/1560-7917.ES.2025.30.1.250109e

**Published:** 2025-01-09

**Authors:** Eurosurveillance editorial team

**Affiliations:** 1 European Centre for Disease Prevention and Control (ECDC), Stockholm, Sweden

In the article entitled ‘Direct and indirect effects of the COVID-19 pandemic on mortality: an individual-level population-scale analysis using linked electronic health records, Wales, United Kingdom, 2016 to 2022’ by Owen et al. published on 12 December 2024, the title of Figure 4G was incorrect in the originally published version. This was corrected on 13 December 2024 and we apologise for any inconvenience this error may have caused.

