# Supplementary material for: Direct and indirect effects of the COVID-19 pandemic on mortality: an individual-level population-scale analysis using linked electronic health records, Wales, United Kingdom, 2016 to 2022
Source: Euro Surveill. 2024 Dec 12;29(50):2400085. doi: 10.2807/1560-7917.ES.2024.29.50.2400085 (PMC11650509; doi:10.2807/1560-7917.ES.2024.29.50.2400085)
Supplement: Supplementary Material [file 24-00085_OWEN_Supplementary_Material.pdf]

Supplementary Material

This supplementary material is hosted by *Eurosurveillance* as supporting information alongside the article 'Direct and indirect effects of the COVID-19 pandemic on mortality: an individual-level population-scale analysis using linked electronic health records, Wales, United Kingdom, 2016 to 2022, on behalf of the authors, who remain responsible for the accuracy and appropriateness of the content. The same standards for ethics, copyright, attributions and permissions as for the article apply. Supplements are not edited by *Eurosurveillance* and the journal is not responsible for the maintenance of any links or email addresses provided therein.

Supplementary Figure S1: All-cause and COVID-specific mortality rate per 100,000 person-months, Wales, 2016 – 2022, (n=6,684,790)

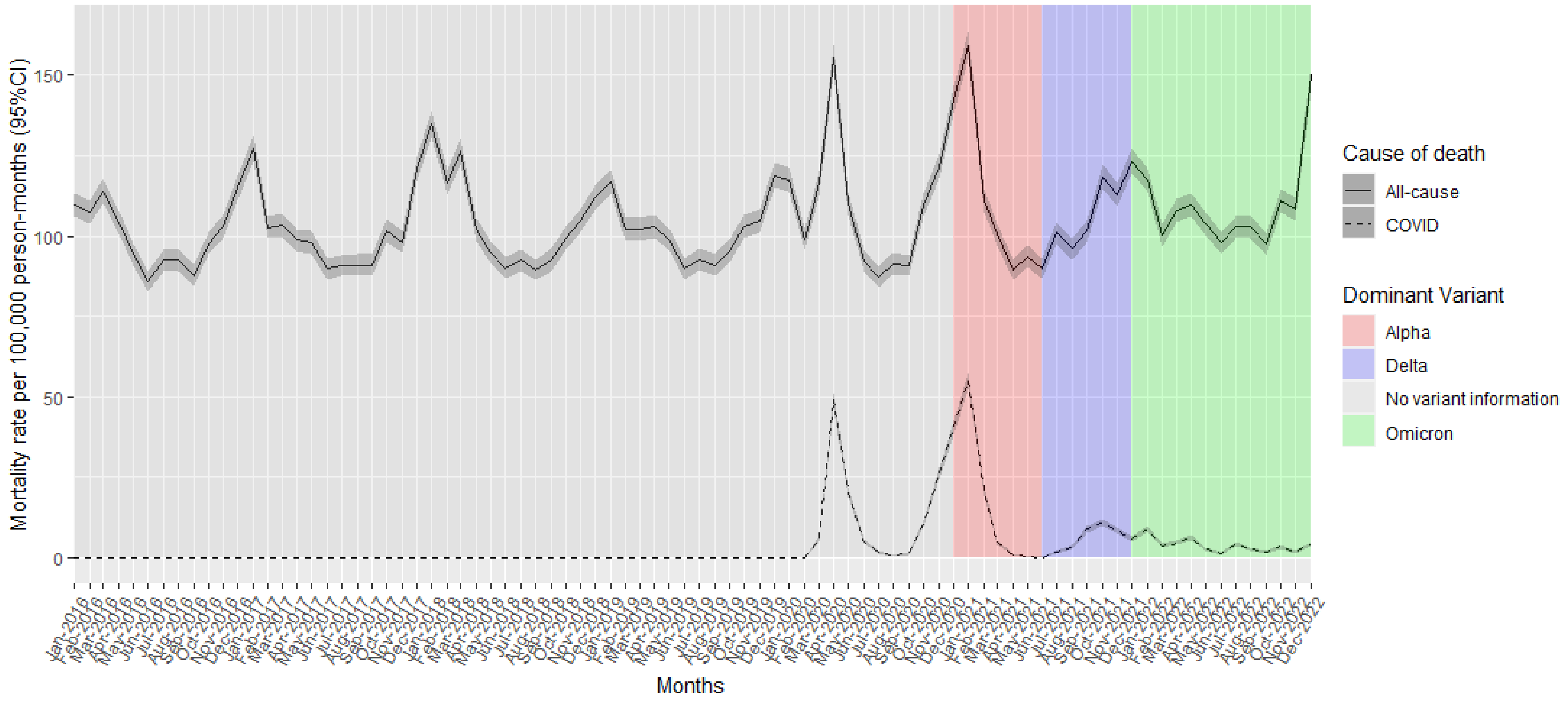

Supplementary Figure S2: All-cause mortality rate per 100,000 person-months by deprivation status, Wales, 2016 – 2022, (n=6,422,388)

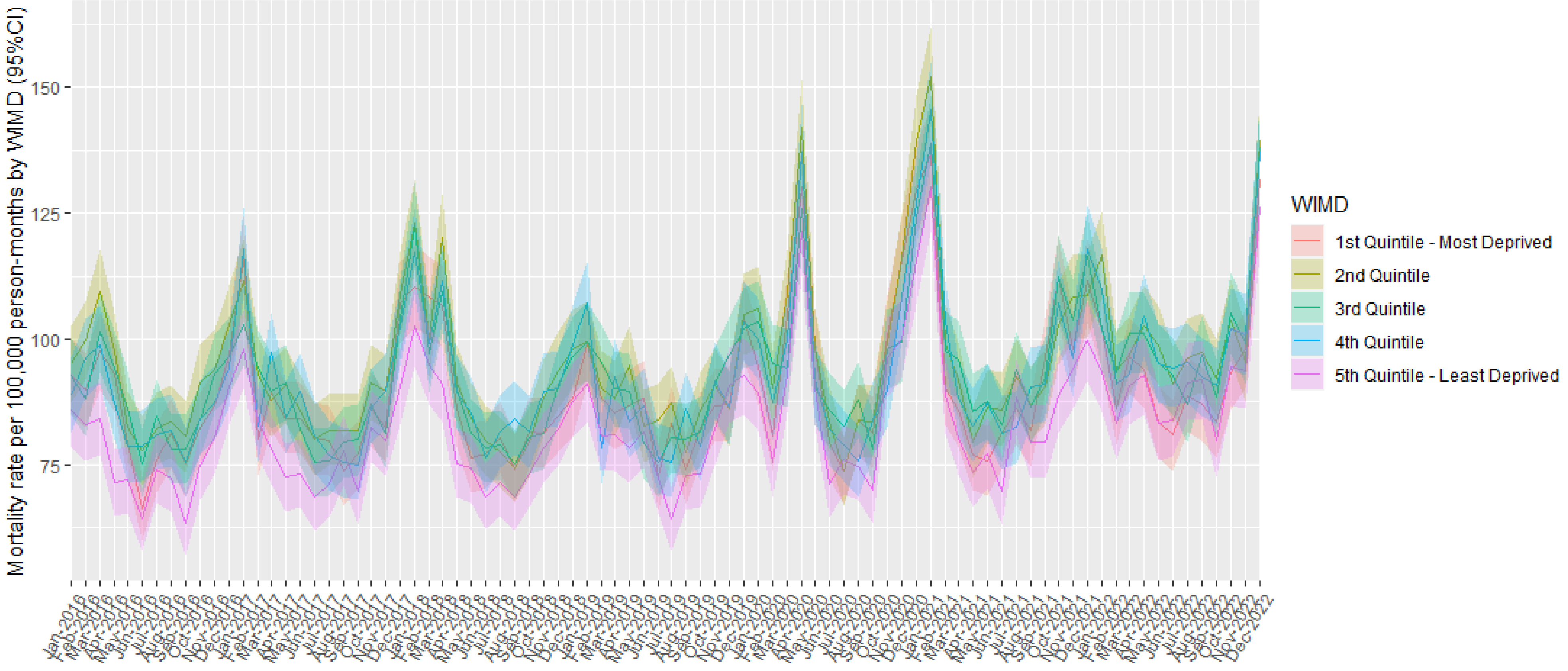

Supplementary Table S1: Relative risks and 95% confidence intervals for all-cause mortality from zero-inflated Poisson regression models including cohort, age, sex, WIMD and the interaction between cohort and WIMD, Wales, 2016 – 2022, (n=6,422,388)

	Relative risk (RR)	95% CI
Intercept	0.004	(0.003, 0.004)
Main effects		
Cohort: C16	Ref	Ref
Cohort: C20	1.07	(1.05, 1.09)
Age	1.106	(1.105, 1.106)
Sex: Male	Ref	Ref
Sex: Female	0.74	(0.74, 0.75)
WIMD 1: Most Deprived	Ref	Ref
WIMD 2	0.87	(0.85, 0.88)
WIMD 3	0.76	(0.74, 0.77)
WIMD 4	0.69	(0.68, 0.71)
WIMD 5: Least Deprived	0.61	(0.60, 0.62)
Interaction effects		
C20*WIMD 1: Most Deprived	Ref	Ref
C20*WIMD 2	0.98	(0.95, 1.00)
C20*WIMD 3	0.97	(0.94, 0.99)
C20*WIMD 4	0.96	(0.93, 0.98)
C20*WIMD 5: Least Deprived	0.97	(0.94, 0.99)

Supplementary Table S2: Relative risks and 95% confidence intervals for all-cause mortality including adults aged 18+ only obtained from zero-inflated Poisson regression models including cohort, age, sex, WIMD and the interaction between cohort and WIMD, Wales, 2016 – 2022, (n=5,099,132)

	Relative risk (RR)	95% CI
Intercept	0.004	(0.003, 0.004)
Main effects		
Cohort: C16	Ref	Ref
Cohort: C20	1.07	(1.05, 1.09)
Age	1.106	(1.105, 1.106)
Sex: Male	Ref	Ref
Sex: Female	0.74	(0.74, 0.75)
WIMD 1: Most Deprived	Ref	Ref
WIMD 2	0.87	(0.85, 0.88)
WIMD 3	0.76	(0.74, 0.77)
WIMD 4	0.69	(0.68, 0.71)
WIMD 5: Least Deprived	0.61	(0.60, 0.62)
Interaction effects		
C20*WIMD 1: Most Deprived	Ref	Ref
C20*WIMD 2	0.98	(0.95, 1.00)
C20*WIMD 3	0.97	(0.94, 0.99)
C20*WIMD 4	0.96	(0.93, 0.98)
C20*WIMD 5: Least Deprived	0.97	(0.94, 0.99)

Supplementary Figure S3: Observed versus expected number of deaths due to falls obtained from negative binomial regression models adjusted for trend and seasonality aggregated by month, Wales, 2016 – 2022 (n=2,597)

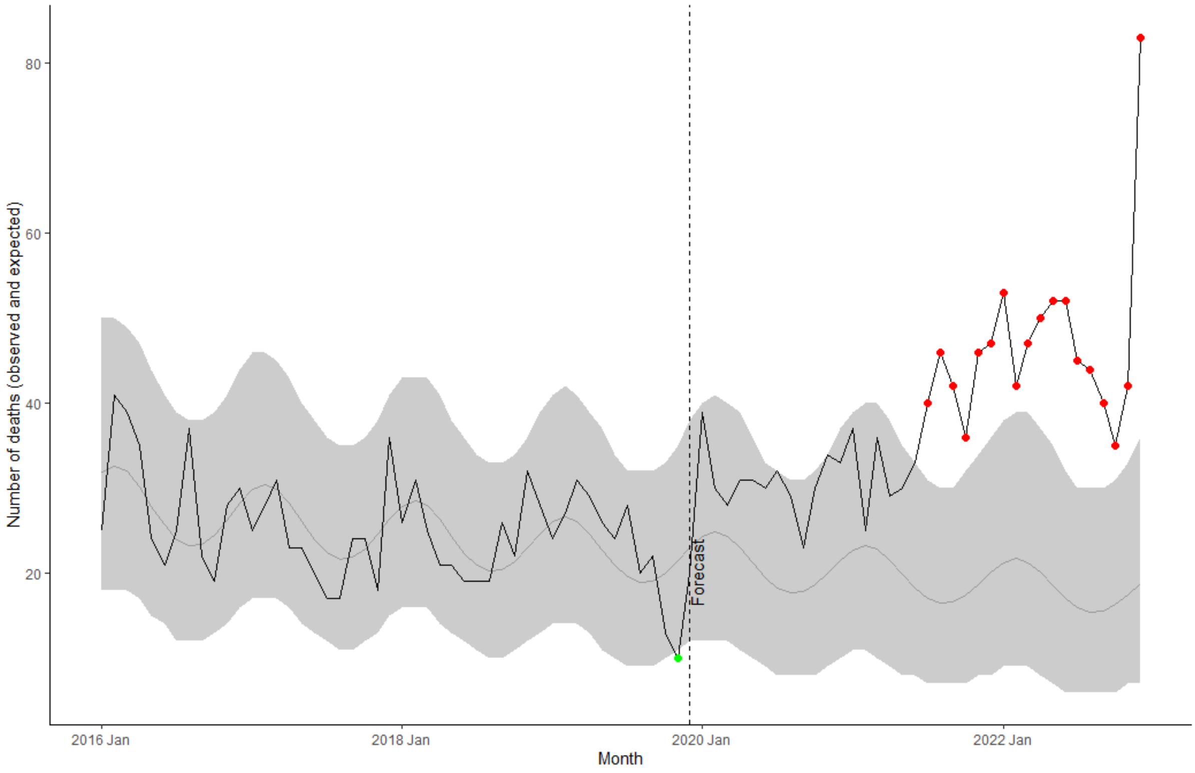

Supplementary Figure S4: Observed versus expected number of deaths due to intent to self-harm obtained from negative binomial regression models adjusted for trend and seasonality aggregated by month, Wales, 2016 – 2022 (n=1,848)

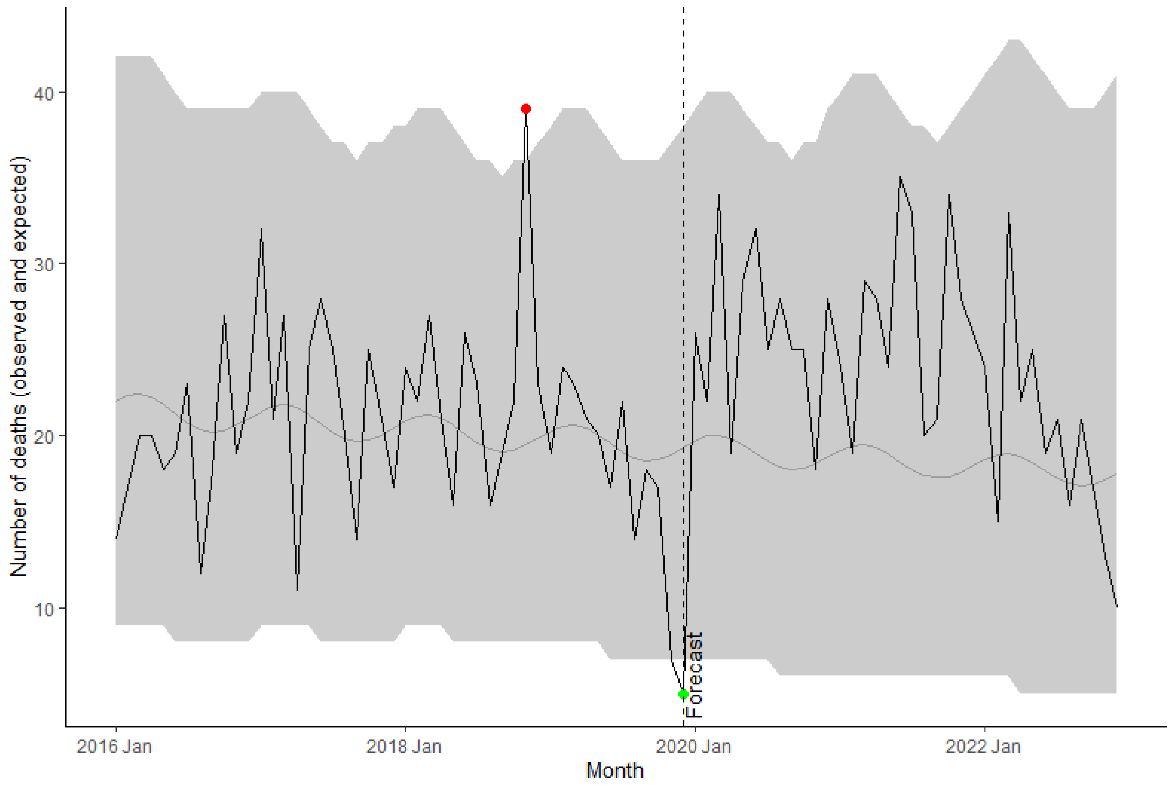

Supplementary Figure S5: Predicted probabilities obtained from multinomial regression models adjusted for age, sex and area-level deprivation using WIMD, Wales, 2016 – 2022 (n=6,422,388)

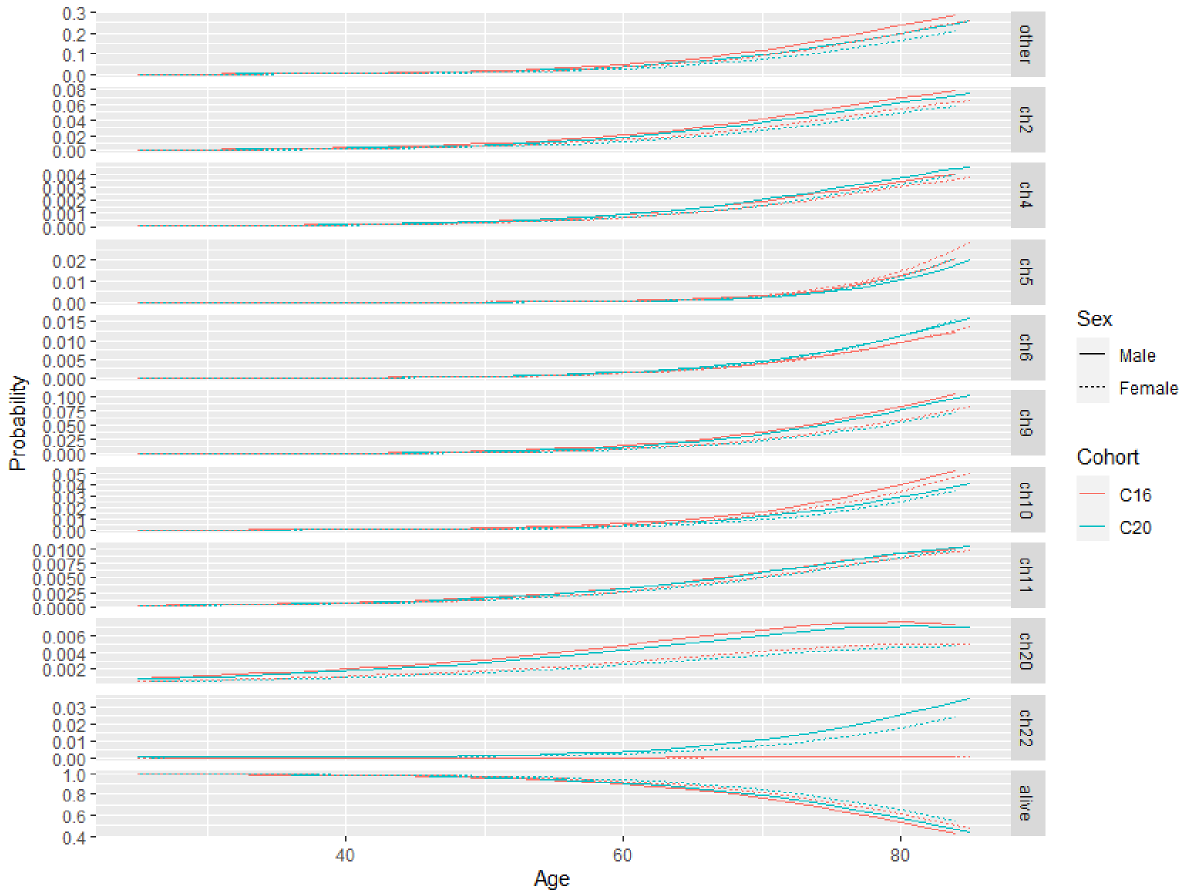

Ch2, Chapter 2: Neoplasms; Ch4, Chapter 4: Endocrine, nutritional & metabolic diseases; Ch5, Chapter 5: Mental & behavioural disorders; Ch6, Chapter 6: Diseases of nervous system; Ch9, Chapter 9: Diseases of circulatory system; Ch10, Chapter 10: Diseases of respiratory system; Ch11, Chapter 11: Diseases of digestive system; Ch20, Chapter 20: External Causes, Ch22, Chapter 22: Special purposes.

Supplementary Table S3: Odds ratios (95% CI) for disease-specific mortality obtained from multinomial regression models adjusted for age, sex and area-level deprivation using WIMD, Wales, 2016 – 2022 (n=6,422,388)

Chapter	Intercept	Cohort: C20	Age	Sex: Female	WIMD: 2	WIMD: 3	WIMD: 4	WIMD: 5 Least Deprived
Other	Ref	Ref	Ref	Ref	Ref	Ref	Ref	Ref
Ch 2: Neoplasms	0.46 (0.45, 0.48)	1.11 (1.09, 1.13)	0.981 (0.981, 0.982)	0.95 (0.93, 0.97)	1.06 (1.03, 1.09)	1.11 (1.08, 1.14)	1.14 (1.11, 1.17)	1.16 (1.13, 1.20)
Ch 4: Endocrine, nutritional & metabolic diseases	0.02 (0.02, 0.03)	1.31 (1.23, 1.39)	0.989 (0.987, 0.991)	1.03 (0.97, 1.10)	0.94 (0.85, 1.03)	0.88 (0.80, 0.96)	0.89 (0.81, 0.98)	0.82 (0.74, 0.90)
Ch 5: Mental & Behavioural disorders	0.005 (0.0048, 0.0058)	0.94 (0.92, 0.97)	1.079 (1.077, 1.081)	1.37 (1.33, 1.41)	1.09 (1.04, 1.14)	1.03 (0.98, 1.08)	1.06 (1.01, 1.11)	1.15 (1.09, 1.20)
Ch 6: Diseases of nervous system	0.02 (0.02, 0.03)	1.38 (1.33, 1.43)	1.02 (1.01, 1.02)	1.19 (1.15, 1.24)	1.17 (1.10, 1.24)	1.23 (1.16, 1.30)	1.36 (1.28, 1.44)	1.65 (1.56, 1.75)
Ch 9: Diseases of circulatory system	0.28 (0.27, 0.29)	1.11 (1.09, 1.13)	1.007 (1.006, 1.008)	0.86 (0.84, 0.87)	1.01 (0.99, 1.04)	1.04 (1.01, 1.07)	1.03 (1.00, 1.05)	0.98 (0.95, 1.01)
Ch 10: Diseases of respiratory system	0.11 (0.10, 0.11)	0.88 (0.86, 0.90)	1.020 (1.019, 1.022)	1.02 (0.99, 1.04)	0.91 (0.88, 0.94)	0.87 (0.84, 0.90)	0.81 (0.78, 0.84)	0.76 (0.73, 0.79)
Ch 11: Diseases of digestive system	0.09 (0.09, 0.10)	1.22 (1.18, 1.27)	0.974 (0.973, 0.975)	1.11 (1.07, 1.15)	0.94 (0.89, 0.99)	0.88 (0.83, 0.92)	0.87 (0.82, 0.92)	0.83 (0.79, 0.88)
Ch20: External causes	0.17 (0.16, 0.19)	1.11 (1.06, 1.15)	0.94 (0.94, 0.95)	0.80 (0.77, 0.83)	0.99 (0.93, 1.05)	0.98 (0.92, 1.05)	0.96 (0.90, 1.02)	0.95 (0.89, 1.02)
Ch 22: Special purposes	0.0008 (0.0007, 0.0010)	112.46 (91.95, 137.55)	1.014 (1.01, 1.02)	0.83 (0.80, 0.87)	0.94 (0.88, 1.00)	0.77 (0.72, 0.82)	0.77 (0.72, 0.83)	0.83 (0.78, 0.88)
Alive	38.37 (37.65, 39.09)	1.29 (1.28, 1.30)	0.899 (0.898, 0.900)	1.39 (1.37, 1.40)	1.23 (1.21, 1.25)	1.47 (1.45, 1.49)	1.64 (1.61, 1.66)	1.89 (1.86, 1.91)

Supplementary Table S4: Odds ratios (95% CI) for disease-specific mortality including adults aged 18+ only obtained from multinomial regression models adjusted for age, sex and area-level deprivation using WIMD, Wales, 2016 – 2022 (n=5,099,132)

Chapter	Intercept	Cohort: C20	Age	Sex: Female	WIMD: 2	WIMD: 3	WIMD: 4	WIMD: 5 Least Deprived
Other	Ref	Ref	Ref	Ref	Ref	Ref	Ref	Ref
Ch 2: Neoplasms	0.48 (0.46, 0.49)	1.11 (1.09, 1.12)	0.980 (0.979, 0.981)	0.95 (0.93, 0.97)	1.06 (1.03, 1.09)	1.11 (1.08, 1.14)	1.15 (1.11, 1.18)	1.17 (1.14, 1.20)
Ch 4: Endocrine, nutritional & metabolic diseases	0.02 (0.02, 0.03)	1.31 (1.23, 1.40)	0.989 (0.987, 0.992)	1.02 (0.96, 1.09)	0.94 (0.86, 1.03)	0.89 (0.81, 0.98)	0.88 (0.80, 0.97)	0.83 (0.75, 0.92)
Ch 5: Mental & Behavioural disorders	0.005 (0.0048, 0.0056)	0.94 (0.92, 0.97)	1.078 (1.077, 1.080)	1.37 (1.33, 1.42)	1.09 (1.04, 1.14)	1.03 (0.99, 1.08)	1.08 (1.03, 1.13)	1.16 (1.11, 1.22)
Ch 6: Diseases of nervous system	0.018 (0.017, 0.020)	1.37 (1.32, 1.42)	1.018 (1.017, 1.020)	1.19 (1.15, 1.23)	1.16 (1.09, 1.23)	1.23 (1.15, 1.30)	1.38 (1.31, 1.47)	1.68 (1.58, 1.78)
Ch 9: Diseases of circulatory system	0.28 (0.27, 0.29)	1.11 (1.09, 1.13)	1.007 (1.006, 1.007)	0.86 (0.85, 0.88)	1.01 (0.98, 1.04)	1.04 (1.02, 1.07)	1.03 (1.00, 1.06)	0.99 (0.96, 1.02)
Ch 10: Diseases of respiratory system	0.11 (0.10, 0.11)	0.88 (0.86, 0.90)	1.020 (1.019, 1.021)	1.02 (0.99, 1.04)	0.92 (0.89, 0.95)	0.88 (0.85, 0.91)	0.81 (0.79, 0.84)	0.77 (0.74, 0.80)
Ch 11: Diseases of digestive system	0.10 (0.09, 0.10)	1.22 (1.17, 1.26)	0.972 (0.971, 0.973)	1.12 (1.08, 1.16)	0.94 (0.89, 0.99)	0.89 (0.84, 0.94)	0.89 (0.84, 0.94)	0.85 (0.80, 0.89)
Ch20: External causes	0.18 (0.17, 0.20)	1.12 (1.07, 1.17)	0.943 (0.942, 0.944)	0.79 (0.76, 0.83)	1.00 (0.94, 1.06)	0.98 (0.92, 1.05)	1.00 (0.94, 1.07)	0.94 (0.88, 1.07)
Ch 22: Special purposes	0.0008 (0.0006, 0.0010)	120.35 (97.71, 148.25)	1.01 (1.01, 1.02)	0.82 (0.79, 0.86)	0.92 (0.87, 0.98)	0.79 (0.74, 0.84)	0.78 (0.73, 0.83)	0.83 (0.78, 0.89)
Alive	39.13 (38.40, 39.88)	1.29 (1.28, 1.30)	0.898 (0.897, 0.898)	1.39 (1.38, 1.40)	1.23 (1.21, 1.25)	1.48 (1.46, 1.50)	1.65 (1.63, 1.67)	1.90 (1.87, 1.93)
